# Supplementary material for: A genome-phenome association study in native microbiomes identifies a mechanism for cytosine modification in DNA and RNA
Source: eLife. 2021 Nov 8;10:e70021. doi: 10.7554/eLife.70021 (PMC8670742; doi:10.7554/eLife.70021)
Supplement: Source data 1. [file elife-70021-supp3.zip › source_data_1/Source data list.docx]

**Source data list**

**Fasta files of modified and unmodified contigs as defined in Figure 3c:**

Figure_3_source_data_1_modified_contigs.fa

**Enrichment score of contigs as plotted in Figure 3c-d:**

Figure_3_source_data_3_enrichment_scores.csv

**Raw and labeled gel pictures shown in supplement Figure 5b:**

Figure_supplement_5_source_data_1_labeled_gel.png

Figure_supplement_5_source_data_1_gel.tif

Figure_supplement_5_source_data_2_labeled_gel.png

Figure_supplement_5_source_data_2_gel.tif
