# Supplementary figures and images for: A genome-phenome association study in native microbiomes identifies a mechanism for cytosine modification in DNA and RNA
Source: eLife. 2021 Nov 8;10:e70021. doi: 10.7554/eLife.70021 (PMC8670742; doi:10.7554/eLife.70021)

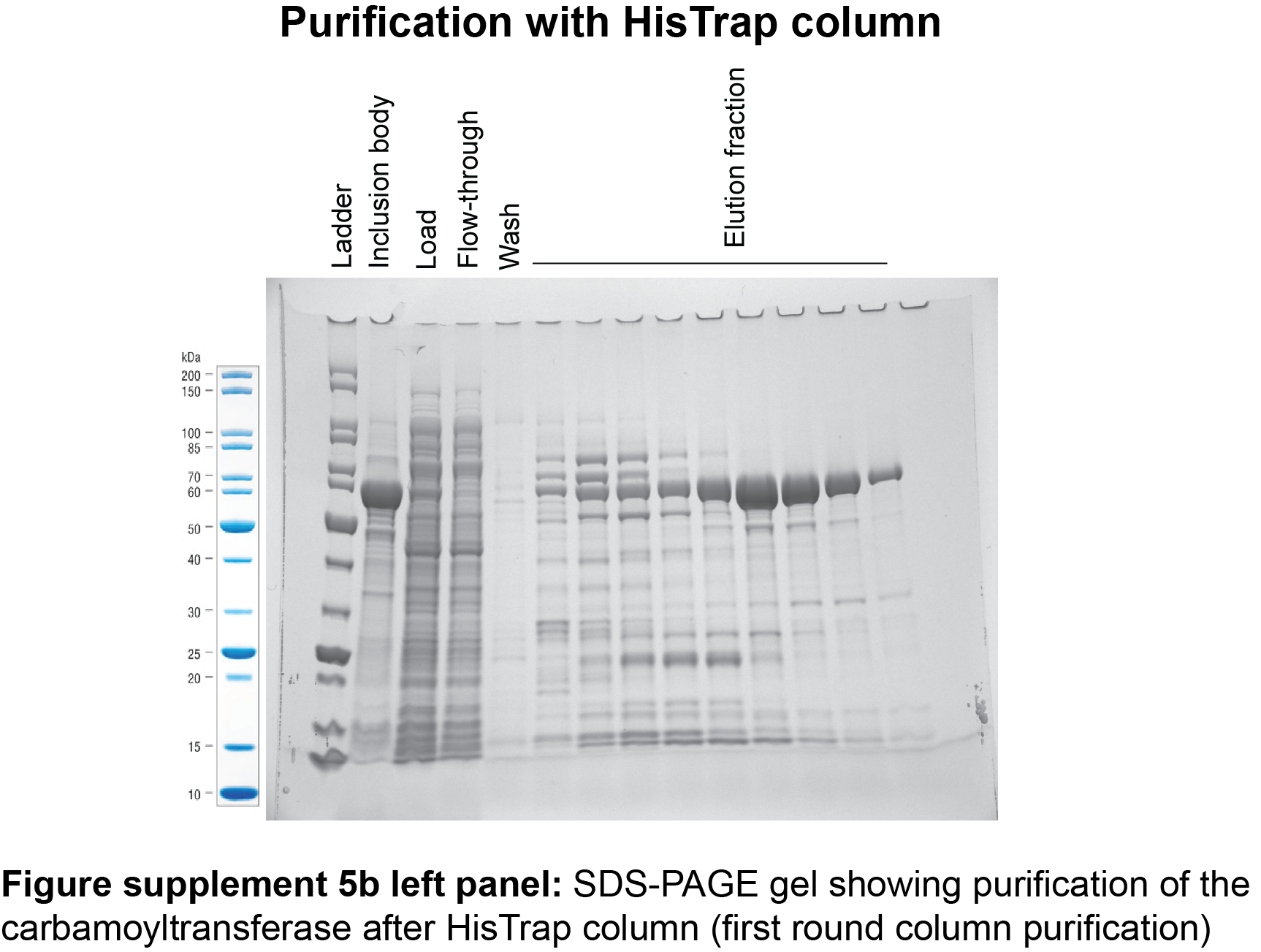

Supplement: Source data 1. [file elife-70021-supp3.zip › source_data_1/Figure_supplement_5_source_data_1_labeled_gel.png]

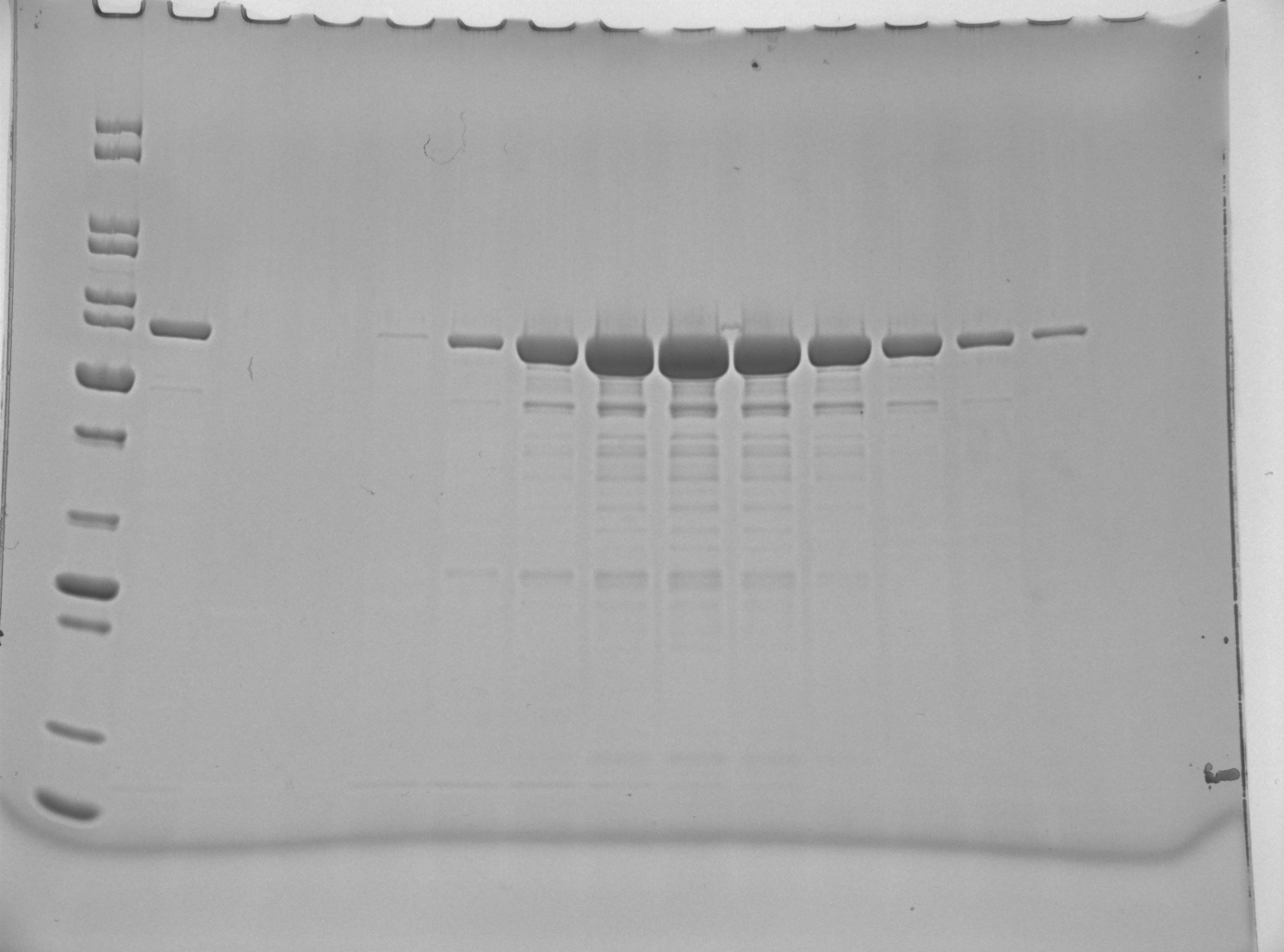

Supplement: Source data 1. [file elife-70021-supp3.zip › source_data_1/Figure_supplement_5_source_data_2_raw_gel.tiff]

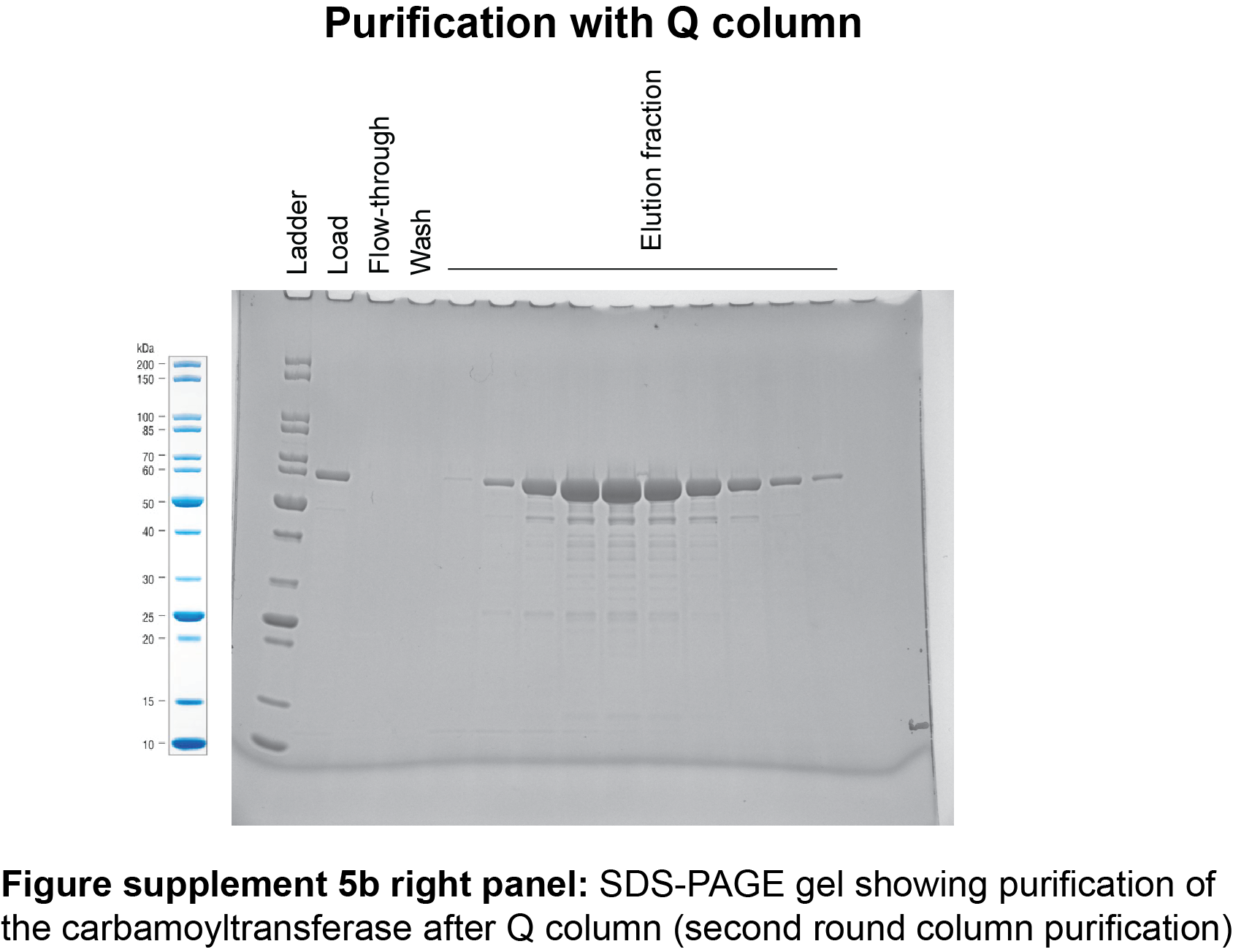

Supplement: Source data 1. [file elife-70021-supp3.zip › source_data_1/Figure_supplement_5_source_data_2_labeled_gel.png]

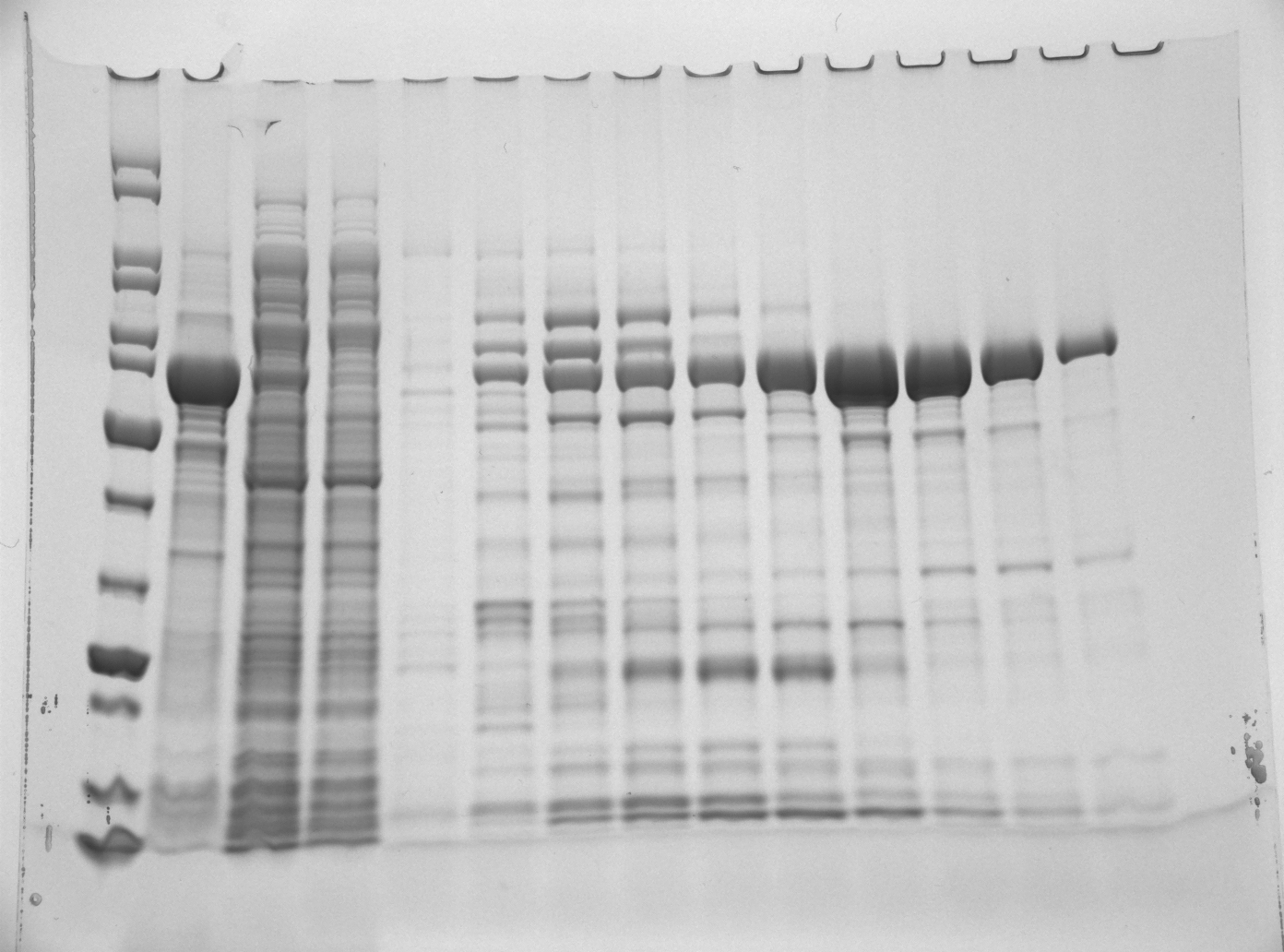

Supplement: Source data 1. [file elife-70021-supp3.zip › source_data_1/Figure_supplement_5_source_data_1_raw_gel.tiff]
